# Phytochemical Compounds, Acute Toxicity, Anti-Inflammatory and Antioxidant Activities of *Thymus leptobotrys* Murb Essential Oil

**DOI:** 10.3390/molecules28031355

**Published:** 2023-01-31

**Authors:** Asmaa Oubihi, Fatima Zahrae Ballaoui, Hamada Imtara, Hassna Jaber, Abdessamad Ettouil, Sara Haida, Mohammed Ouhssine, Omar M. Noman, Ramzi A. Mothana, Mahmoud Tarayrah, Zineb Guessous

**Affiliations:** 1 Laboratory of Natural Resources and Sustainable Development, Faculty of Science, University Ibn Tofail, Kenitra B.P 242, Morocco; 2 Pharmacodynamy Research Team ERP, Laboratory of Pharmacology and Toxicology, Faculty of Medicine and Pharmacy, University Mohammed V, Rabat B.P: 8007.N.U, Morocco; 3 Faculty of Arts and Sciences, Arab American University Palestine, Jenin P.O. Box 240, Palestine; 4 Laboratory of Separation Processes, Team of Environment and Applied Chemistry, Faculty of Sciences, Ibn Tofail University, Kenitra B.P 242, Morocco; 5 Department of Pharmacognosy, College of Pharmacy, King Saud University, P.O. Box 2457, Riyadh 11451, Saudi Arabia; 6 Groupe Hospitalier Cochin-Port Royal, Faculty of Medicine, Institut Cochin, Paris University, CNRS, IN-SERM, 75000 Paris, France

**Keywords:** *Thymus leptobotrys Murb*, essential oil, acute toxicity, anti-inflammatory effect, antioxidant activity

## Abstract

The present study was conducted to evaluate the acute toxicity and anti-inflammatory effect in vivo, as well as the antioxidant activity, of the essential oil of *Thymus leptobotrys Murb*. The results indicate that the tested essential oil is non-toxic, with an estimated LD50 of 2500 mg kg^−1^ of mice body weight. The anti-inflammatory test revealed that, at a dose of 200 mg kg^−1^, the essential oil reduced rat paw edemas by 89.59% within 3 h of oral administration, this reduction in edema size was greater than that obtained with indomethacin (75.78%). The antioxidant activity (IC50) of *Thymus leptobotrys Murb* essential oil was 346.896 µg mL^−1^ and 861.136 mg Trolox equivalent/g essential oil in the 2.2-diphenyl1-picryl-hydrazyl radical scavenging capacity (DPPH) and Trolox equivalent antioxidant capacity (TEAC) assays, respectively. The toxicity test reveals an LD_50_ greater than 2500 mg kg^−1^ of body weight of mice which classifies it within category 5 of non-toxic substances that can be administered orally. These results suggest that the essential oil of *Thymus leptobotrys Murb* is not toxic, and it represents a valuable source of anti-inflammatory and antioxidant metabolites.

## 1. Introduction

Inflammation is recognised today as a silent killer, responsible for many acute and chronic diseases such as rheumatoid arthritis, diabetes, atherosclerosis, obesity, and cancer [1,2,3]. Current treatment of inflammation is based on the use of steroidal and non-steroidal anti-inflammatory drugs. These drugs are effective, but their prolonged use can cause various adverse effects, i.e., malaise, gastrointestinal, liver or kidney toxicity, and inhibition of platelet aggregation [4,5]. Lipopolysaccharide (LPS) can activate monocytes, neutrophils, and macrophages during inflammatory reactions, causing excessive production of numerous pro-inflammatory and toxicity-mediated molecules such as tumor necrosis factor (TNF-), interleukin-6 (IL-6), eicosanoids, and nitric oxide (NO) [6]. NO and prostaglandins (PG) are two major pro-inflammatory mediators [1,7]. Inhibition of PG and NO production is achieved via inhibition of their synthases that is, cyclooxygenase 2 (COX-2) and inducible nitric oxide synthase (iNOS) [8]. The first line of clinical treatment for inflammatory disorders relies on non-steroidal anti-inflammatory drugs (NSAIDs), but steroidal agents and immunosuppressants are also used [9]. Indomethacin is one of the NSAIDs commonly used in the treatment of inflammation. It is a well-established fact that NSAIDs exert their anti-inflammatory activity by inhibiting prostaglandin biosynthesis. However, the use of anti-inflammatory drugs, non-steroidal (NSAIDs) or steroidal, is associated with adverse effects, such as gastrointestinal discomfort, inhibition of platelet aggregation, and liver and kidney toxicity [4,5]. Moreover, the human body is protected against many diseases by antioxidants that act as free radical scavengers [10]. The danger of free radicals comes from the damage they can cause when they react with important cellular components such as DNA, lipids, and proteins [11,12]. This oxidation causes damage throughout the body, namely degenerative diseases, cancer, premature ageing, and cardiovascular diseases [13]. Therefore, the further search for alternative drugs remains inevitable, although the use of natural product-based compounds with satisfying therapeutic effects and little or no side effects is increasingly proving to be a necessity.

Medicinal plants are an inexhaustible source of molecules with a wide range of biological and pharmacological activities. Numerous studies have shown that plants, especially their essential oils, have antioxidant and anti-inflammatory properties due in large part to their phenolic compounds [9,14,15]. Most of the chemical constituents of essential oils are terpenoids, characterised by a low molecular weight which allows easy transport across cell membranes to induce different biological activities, including anti-inflammatory and antioxidant effects [16,17,18]. Studies have also shown that carvacrol, a phenolic compound found in several essential oils, can effectively protect cells and tissues from oxidative damage, thus making it a potent antioxidant [19,20,21]. Regarding its anti-inflammatory properties, carvacrol has been shown to modulate the activity of various inflammatory mediators, such as the production of pro-inflammatory cytokines and the expression of adhesion molecules [22,23,24].

Among many endemic plants of the Moroccan flora, one essential oil of particular interest is that of *Thymus leptobotrys* Murb, known for a high percentage of carvacrol and widely used in traditional medicine. *Thymus leptobotrys* Murb is a Moroccan endemic species of the Lamiaceae family, which are perennial, aromatic herbs widely used in the Mediterranean basin [25]. Traditional medicine has used *Thymus leptobotrys* Murb as a remedy in the form of powders, infusions, and decoctions for the treatment of many ailments, including whooping cough, rheumatism, bronchitis, and indigestion [26,27]. In addition, extracts of *Thymus leptobotrys* Murb are reported to possess potent antibacterial [28,29,30], antifungal [31,32], analgesic [33], antioxidant [34,35], and insecticidal activities [36].

Many studies have shown the biological properties of *Thymus leptobotrys* Murb plant extracts. In our previous study, we evaluated the anti-inflommatory activity of *Thymus leptobotrys* Murb extracts by using carrageenan method. However, to our knowledge, an evaluation of the anti-inflammatory activity of the essential oil of this plant has not been reported in the literature to date. In this context, the objective of the present work is to study the anti-inflammatory effect in vivo as well as the acute toxicity and antioxidant power of the essential oil of *Thymus leptobotrys* Murb.

## 2. Results and Discussion

### 2.1. Chemical Composition

GC-MS analysis identified 31 constituents, representing about 99.31% of the total composition of *T. leptobotrys* essential oil (Table 1). Oxygenated monoterpenes are the most common group in the essential oil, of which carvacrol (73.68%) is the major compound, followed by the two monoterpene hydrocarbons, p-cymene (8.68%) and γ-terpinene (4.14%). Other compounds are also present, but at low levels, such as α-pinene (2.84%), β-caryophyllene (2.51%), and terpinene-4-ol (1.14%). These six main components among the 31 identified represent 92.99% of the essential oil. The secondary components represent 6.32%. These results are consistent with many studies, as carvacrol is the main compound present in the essential oil of this plant, as it was clarified in our previous study [30].

These results are also consistent with the study conducted by Hamada et al on the Origanum vulgare plant, where the study showed that carvacrol and thymol are the two main components in the essential oil extracted from that plant [37], but the percentage of the carvacrol compound was less than that found in the essential oil extracted from Thymus leptobotrys Murb.

### 2.2. Acute Toxicity

**Figure 1:**
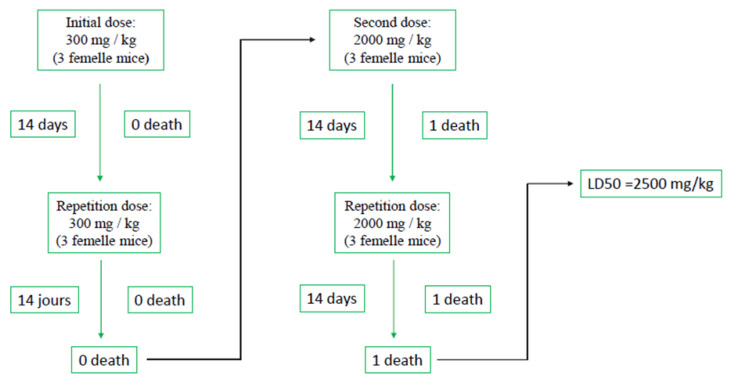
Determination of the LD_50_ of *Thymus leptobotrys* Murb essential oil.

**Figure 2:**
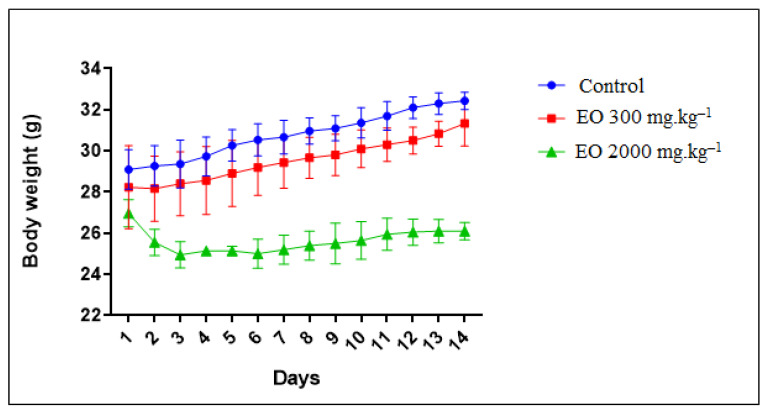
Evolution of the average weight of mice treated with orally administered *Thymus leptobotrys* Murb essential oil (EO) as a function of time in days. The data are represented as means ± SD.

The acute toxicity of the essential oil of *T. leptobotrys* was assessed according to the Organization for Economic Co-operation and Development (OECD) Guideline 423. The results are summarised in Figure 1 and Figure 2.

The administration of *T. leptobotrys* essential oil at a dose of 2000 mg EO.kg^−1^ body weight caused some signs of toxicity after one hour; a sedative effect with disturbed breathing followed by the prostration of the mice was observed. The mice did not return to normal until the third day. Only one case of death was recorded on the second day (Figure 1). The surviving mice showed a lack of appetite with weight loss. Following these results and the case of death, and according to the OECD guideline 423, the same dose (2000 mg EO.kg^−1^) was administered a second time to other healthy mice to confirm the results, the so-called confirmation dose (Figure 1). In the second case, one mouse died again, and the survivors showed the same effects as the survivors of the first batch. We can thus establish the LD_50_ of *T. leptobotrys* essential oil to be 2500 mg EO.kg^−1^ body weight, which allows us to classify this essential oil, according to the OECD, in category 5 of non-toxic substances by the oral route; as a similar LD_50_ (2.66 g.kg^−1^) was reported [38].

These higher LD_50_ value indicates a lower toxicity, which supports the use of these extracts and essential oils for appropriate safety. According to Clarke et al. [39]. compounds with a LD_50_ of 1000 mg kg^−1^ of body weight by oral ingestion suggests that it would take a relatively high dose of the substance to cause death in 50% of the test subjects, this may indicate that the compound has considered to be relatively safe or have low toxicity. This may be explained by the fact that the substance may be detoxified during liver passage or by a lower absorption of the substance through the gastrointestinal tract [40].

Nevertheless, mice treated with *T. leptobotrys* essential oil showed signs of sedative at higher doses, which include drowsiness, calmness, and a reduction in anxiety and agitation in their movements during the first hours after the oral administration. This demonstrates that the essential oil of *T. leptobotrys* has a sedative effect on the nervous system of the mice at high doses. Therefore, further studies would be needed to confirm the sedative effects of *T. leptobotrys* essential oil in humans and for understanding the potential therapeutic benefits of *T. leptobotrys* essential oil.

Monitoring of the weight evolution of mice treated with *T. leptobotrys* essential oil at a dose of 300 mg EO.kg^−1^ led to the observation of stability of body weight for 14 days (Figure 2). On the other hand, at a dose of 2000 mg EO.kg^−1^ of body weight, a rapid and significant decrease in the weight of the mice was observed, which was stabilised from the 2nd day onwards (Figure 2). This is the first time to our knowledge that such an in vivo study has been carried out. The absence of bibliographic data does not allow us to explain this toxic effect with certainty, but we assume that the majority of carvacrol in the essential oil (73.68%) was responsible for this effect and is toxic at this dose.

### 2.3. Anti-Inflammatory Activity

**Figure 3:**
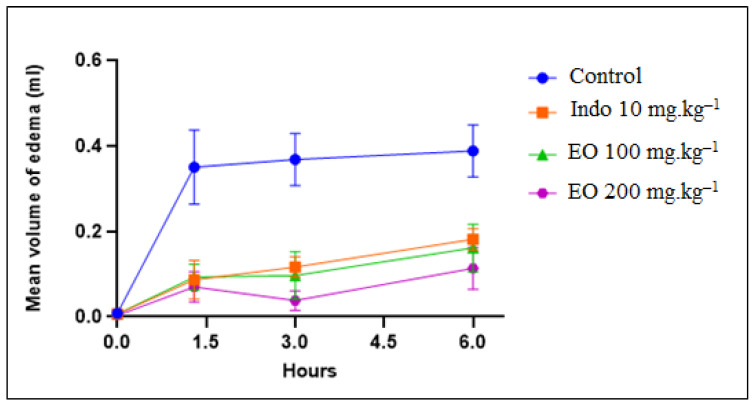
Influence of *Thymus leptobotrys* Murb essential oil (100 and 200 mg kg^−1^) on carrageenan-induced edema. Data represent the difference in mean edema volume (mean ± S.E.M), *p* ≤ 0.001 compared to the control. Indo: Indomethacin; EO: Essential oil.

Figure 3 shows the effect of *T. leptobotrys* essential oil on the evolution of carrageenan-induced edemas on the posterior left paw of rats (*n* = 6) as a function of time. Subplantar injection of 1% carrageenan to the control group resulted in a significant increase in the mean volume of the edema to 0.35, 0.368, and 0.388 mL at 1.30, 3, and 6 h, respectively (Figure 3). This provided evidence that the injection of carrageenan into the rat paw caused a local and acute inflammatory response [41,42].

Indomethacin was used as a standard anti-inflammatory drug because it has been widely used to exhibit anti-inflammatory properties, and its action mechanisms are well-understood. It also improves the validity and reliability of our results by providing points of reference for the anti-inflammatory effects of the essential oil. In our study, the administration of indomethacin (10 mg/kg body weight) reduced significantly the increase in left paw volume by 0.086, 0.116, and 0.181 mL at 1.30, 3, and 6 h (Figure 3). 

The anti-inflammatory effect of *T. leptobotrys* essential oil was optimal 3 h after the induction of the edemas. At the concentration of 200 mg EO.kg^−1^ body weight, *T. leptobotrys* essential oil had a significant anti-inflammatory activity, with the percentage of edema inhibition at 89.59% after 3 h (Table 2); the edemas were significantly reduced to 0.038 ± 0.018 (*p* ≤ 0.001) (Figure 3). This inhibition was higher than that of indomethacin (68.32%) (Table 2). Furthermore, although indomethacin is a reference anti-inflammatory, its effect tends to decrease after 6 h, while *T. leptobotrys* essential oil has a stable activity.

The carrageenan-induced paw edemas were due to cyclooxygenase and lipooxygenase [43,44]. The cyclooxygenase enzyme is directly involved in the biosynthesis of prostaglandins, which are key mediators of the inflammation mechanism, while lipoxygenase, while lipoxygenase is involved in inflammation process because of its role in leukotriene biosynthesis [45]. Leukotrienes produced are pro-inflammatory mediators that activate immune cells and increase vascular permeability which modulate inflammation and immunity. A biphasic reaction characterizes the inflammatory phenomena [45]. The first phase, observed at 1:30 min to 2 h, depends on the release of chemical mediators such as histamine, serotonin, and bradykinin. The second phase is the swelling phase (3–6 h), supported by the release of leukotrienes, prostaglandins, lysozymes, proteases, and nitric oxide (NO), cytokines (TNF-α, IL-8, and IL-1β) [46,47,48].

The anti-inflammatory process of *T. leptobotrys* essential oil could be related to the inhibition of the release, or synthesis, of cyclooxygenase products by carvacrol, a component with a phenolic structure, which is attributed to anti-inflammatory properties [49]. The high content of this component in the essential oil of *T. leptobotrys* (73.68%) is probably the reason for its high activity. Other works have suggested that carvacrol inhibits prostaglandin biosynthesis [50,51,52].

### 2.4. Antioxidant Activity

The antioxidant activity of *T. leptobotrys* essential oil was evaluated by two complementary tests: 2.2-diphenyl1-picryl-hydrazyl (DPPH) free radical scavenging and 2.2-azino-bis (3-ethylbenzothiazoline-6-sulfonic acid) (ABTS) radical scavenging capacity (Table 3). A low IC_50_ value indicates significant antioxidant activity. In the 2.2-diphenyl1-picryl-hydrazyl free radical scavenging (DPPH) test, the IC_50_ of *T. leptobotrys* essential oil was in the range of 346.90 ± 0.53 µg mL^−1^; the antioxidant activity of *T. leptobotrys* was lower than that of Trolox (1.85 ± 0.02 µg mL^−1^) which was used as a positive control. The 2.2-azino-bis (3-ethylbenzothiazoline-6-sulfonic acid) (ABTS) radical scavenging capacity (ABTS) test confirmed the high antioxidant capacity (861.14 ± 1.21 mg equivalent Trolox/g essential oil) of the *T. leptobotrys* essential oil.

The antioxidant activity obtained could be due to carvacrol, the major component of the essential oil. Many studies report that the high antioxidant potential of essential oils of several Thymus species could be related to the presence of oxygenated compounds and the nature of its phenolic compounds, such as carvacrol and thymol [53,54]. Other, more specific studies on carvacrol have highlighted its powerful antioxidant activity [55,56]. Phenolic compounds, because of their redox properties, act as hydrogen and singular oxygen donor reducing agents [57]. However, the antioxidant activity of the majority of compounds tested separately gave lower results compared to the activity of the whole essential oil [58]. Synergistic interactions between the different constituents of an essential oil result in a much higher antioxidant capacity [57]. Indeed, some compounds other than phenolics, such as γ-terpinene, also possess high antioxidant activity [59]. Finally, the structural characteristics of these molecules were attributable to the high reactivity of the hydroxyl substituent group [60].

The investigated of *T. leptobotrys* essential oil shows low activity compared to the standard antioxidant Trolox, which has an IC_50_ of 1.810 ± 0.017 µg mL^−1^. However, their antioxidant activity could be improved and probably approach those of the standards by a proper purification method of the active molecules. Further research is needed to better identify, isolate and purify the active components because they have the advantage of being natural-based products since they come from plant sources, unlike synthetic antioxidants which are questionable due to potential health risks.

## 3. Material and Methods

### 3.1. Plant Material

The aerial parts of *T. leptobotrys* Murb were harvested in the region of Sidi Mzal (N 29°86′/W 08°88′) located in the mountains of the Moroccan Anti-Atlas during the flowering period (April–June). Drying was conducted at room temperature. Then, the dried leaves were reduced to powder and saved in sterile sachets for further analysis.

### 3.2. Animals

Adult Wistar rats (180 to 230 g) and adult Swiss mice (20 to 30 g) were used to test the anti-inflammatory activity and acute toxicity. The rats were taken from the animal centre of the Faculty of Medicine and Pharmacy, Mohammed V University of Rabat. The experimental conditions during the maturation of the rats followed the standard conditions: temperature between 20 and 25 °C with a photoperiodic cycle of 12 h. This study complied with the requirements stated in the report “Guide for the care and use of laboratory animals”, produced by the NAS “National Academy of Sciences” and edited by the National Institutes of Health [61].

### 3.3. Extraction of the Essential Oil

The essential oil of *T. leptobotrys* was obtained by hydrodistillation in a Clevenger-type apparatus for 4 h until the total recovery of the oil (2.5% v/p) [30]. The essential oil was dried on anhydrous sodium sulphate and stored in dark glass bottles at +4 °C until use.

### 3.4. Chromatographic Analysis

Chromatographic analyses were carried out using a gas chromatograph (Perkin ElmerTM GC-680) coupled with mass spectrometry (Q-8 MS with ion trap) [30]. Fragmentation was performed by electron impact at a field strength of 70 eV. The capillary column used was an MS Rxi^®^-5sil (1,4-bis (dimethylsiloxy) phenylene dimethyl polysiloxane (30 m × 0.25 mm)), the film thickness was 0.25μm, the column temperature was programmed from 60 to 310 °C at 4 °C/min, the carrier gas was helium with a flow rate of 1 mL.min^−1^, the sample injection was in split mode, and the apparatus was connected to a computer system managing a library of NIST mass spectra.

The components were identified on the basis of their mass spectra obtained by gas chromatography coupled with mass spectrometry (GC-MS) and their Kovat indices (IK).

### 3.5. Acute Oral Toxicity

The acute toxicity of *T. leptobotrys* Murb essential oil was tested on adult Swiss mice according to the guidelines given by the Organization for Economic Co-operation and Development (OCDE) [62]. The body weight of each animal was determined after 4 h of fasting. The doses were expressed as mg of essential oil per kg of body weight. Three groups of three mice were randomly assigned. Two groups were administered the essential oil of *T. leptobotrys* orally at doses of 300 and 2000 mg kg^−1^, and the third group received distilled water (control group). Assessment of signs of toxicity included general ingestion of food and water, behavioural symptoms, convulsions, respiration, changes in body weight, and mortality. These signs were evaluated in a systematic way for each group in the first hours after administration and after 14 days. The 50% lethal dose (LD_50_) was calculated following the protocol given by the 423 guideline [62].

### 3.6. Anti-Inflammatory Activity

The anti-inflammatory activity was evaluated by the carrageenan-induced paw edema approach [63,64]. Before the tests, four groups of rats, with six rats in each group, were subjected to an 18 h fasting period. Concentrations of 100 and 200 mg kg^−1^ of *T. leptobotrys* Murb essential oil were applied to the rat groups by oral administration. The control group and the last group were given with distilled water and the reference drug (10 mg kg^−1^ of indomethacin), respectively. After 30 min, subcutaneous injection of 0.05 mL of 1% carrageenan solution was administered to all groups of rats in the subplantar region of the left hind paw. An LE7500 plethysmometer was used to measure the volume of the rat’s paw at several time points: prior to carrageenan injection (V0), at 1 h 30 min, 3 h, and 6 h following carrageenan injection (Vt). The anti-inflammatory effect was measured as follows [65]:

(1) $$\%\;\mathrm{of}\;\mathrm{inhibition}\;=\frac{\;\left(\mathrm{Vt}-V0\right)\;\mathrm{control}-\left(\mathrm{Vt}-V0\right)\;\mathrm{treated}\;\mathrm{group}}{\left(\mathrm{Vt}-V0\right)\;\mathrm{control}}\times 100$$

### 3.7. Antioxidant Activity

#### 3.7.1. DPPH Free Radical Scavenging

The capacity of essential oil and extracts to scavenge the 2.2-diphenyl1-picryl-hydrazyl (DPPH) was determined according to the protocol described by Sindhu et al. [66]. The DPPH method is generally the most commonly used for the rapid and direct assessment of antioxidant activity due to the stability of the free radicals generated as well as the simplicity of the analysis. A 0.2 mM solution of DPPH was prepared in ethanol and 0.5 mL of this solution was added to 2.5 mL of the sample. After shaking to homogenise the mixture, the mixture was kept for 30 min in the dark [67]. The absorbance was measured at 517 nm. Trolox was used as the reference compound. The ability to recover the DPPH radical was measured as follows:

(2) $$\%\;\mathrm{of}\;\mathrm{inhibition}\;=\frac{\;\left(A0-A1\right)}{A0}\times 100$$

where A0 is the absorbance of the negative control and A1 is the absorbance of the sample.

The trapping activity is expressed by the IC_50_ which represents the concentration of the sample necessary to inhibit 50% of the trapping activity of free radicals.

#### 3.7.2. Trolox Equivalent Antioxidant Capacity (TEAC)

The determination of the antioxidant capacity was performed by 2.2-azino-bis (3-ethylbenzothiazoline-6-sulfonic acid) (ABTS) free radical scavenging [67]. First, stock solutions of 7 mM ABTS and 2.4 mM potassium persulfate (K_2_S_2_O_8_) were prepared in identical volumes and then stored at room temperature for 6 h in the dark. Before measuring the absorbance at 734 nm, the ABTS+ solution was diluted with ethanol to obtain an absorbance of 0.700 ± 0.02 at 734 nm. Then, a reaction between 2 mL of the resulting solutions with 200 µL of the sample (2 mg/mL) was maintained for 30 min. Following the same protocol, the percentage of ABTS+ inhibition by different concentrations of Trolox ranging from 5 to 100 µg mL^−1^ was measured. The antioxidant capacity of the sample was expressed in mg Trolox equivalent per gram of extract (mg TE/g E). The test is carried out in triplicate.

### 3.8. Statistical Analysis

For each measurement, the data were expressed as mean values ± standard deviation, analysed employing analysis of variance (ANOVA One-way) followed by Tukey’s post-test. The statistical study was performed using Graph pad prism 8 software. A probability of *p* < 0.05 indicates that the values were considered statistically significant.

## 4. Conclusions

The present study aimed to investigate the anti-inflammatory, antioxidant activities and the acute toxicity of *Thymus leptobotrys* Murb essential oil. Our results indicate that the essential oil exhibits strong anti-inflammatory activity as well as antioxidant properties.

In the acute oral toxicity test, the essential oil was found to be safe at the tested dosages. In the anti-inflammatory test, the essential oil reduced paw edemas in a dose-dependent manner, similar to the reference drug, indomethacin. In the antioxidant test, the essential oil showed similar antioxidant activity to the reference compound Trolox. Additionally, these findings provide important information for the safe use of the essential oil and opens new possibilities for future research on the therapeutic properties of this essential oil. Despite the promising indications, further research is needed to fully understand the cytotoxicity, cellular uptake efficiency, and mechanism for antioxidant activity of the *Thymus leptobotrys* Murb essential oil. Further supporting studies are needed to establish its safety and efficacy in different populations, as well as to evaluate its activities using in vitro methods.

## Figures and Tables

**Table 1:** Chemical compounds obtained from the essential oil of *Thymus leptobotrys* Murb.

	RT	Compound	%
1	838	2-hexenal	Tr
2	916	3-heptanone	Tr
3	936	α-pinene	2.84
4	980	β-pinene	Tr
5	1025	P-cymene	8.68
6	1047	(E) β-ocimene	0.38
7	1059	γ-terpinene	4.14
8	1067	Sabinene-hydrate	0.05
9	1088	α-terpinolene	0.24
10	1099	Linalol	0.95
11	1122	Trans-p-menth-2-en-1-ol	Tr
12	1123	Pinocarveol	0.14
13	1179	Terpinene-4-ol	1.14
14	1191	Cis-dihydrocarvone	0.15
15	1196	α-terpineol	0.05
16	1204	Dihydrocarvone	0.19
17	1234	Carvacrol methyl ether	0.56
18	1302	Carvacrol	73.68
19	1358	Eugenol	0.23
20	1381	Carvacryl acetate	0.57
21	1426	β-caryophyllene	2.51
22	1445	Aromadendrene	0.91
23	1443	α-guaiene	0.07
24	1467	Alloaromandendrene	0.32
25	1480	β-guaiene	0.05
26	1524	γ-cadinene	0.14
27	1532	δ-cadinene	0.21
28	1587	Spathulenol	0.92
29	1635	β- eudesmol	Tr
30	1638	Epi-α-cadinol	0.19
31	1939	Phytol	Tr
Total identified %			99.31

RT: Retention Time; Tr: Trace amounts (<0.05). %: Percentage

**Table 2:** Percentage decrease in volume of the left hind paw in rats treated with the essential oil of *Thymus leptobotrys* Murb.

Treatments (mg kg^−1^)	Inhibition of Edema Induced by Carrageenan (%)		
	1 h 30 min	3 h	6 h
Indo 10	75.24 ± 0.025	68.32 ± 0.017	53.22 ± 0.018
EO 100	73.33 ± 0.021	73.55 ± 0.028	58.37 ± 0.026
EO 200	80.00 ± 0.030	89.59 ± 0.018	70.81 ± 0.027

EO: essential oil; Indo: Indomethacin

**Table 3:** Antioxidant activity of the essential oil of *Thymus leptobotrys* Murb.

Assays	*T. leptobotrys*	Positive Control
	EO	Trolox
DPPH (IC_50_ µg mL^−1^)	346.90 ± 0.53	1.85 ± 0.02
ABTS (mg Trolox equivalent/g EO)	861.14 ± 1.21	-

EO: essential oil.

## Data Availability

Not applicable.

## Sample Availability

Samples of the compounds are available from the authors.
